# Efficacy of non-steroidal anti-inflammatory drug (NSAID) treatment for bovine respiratory disease: a systematic review and meta-analysis for the European Network for Optimization of Antimicrobial Therapy guidelines

**DOI:** 10.12688/openreseurope.23174.1

**Published:** 2026-03-18

**Authors:** Karolina Scahill, Luis Pedro Carmo, Ramazan Yildiz, Mickael Cargnel, Sébastien Buczinski, Clair L Firth, Maria Stokstad, Pierre-Louis Toutain, Amelia R Woolums, Lise Marie Ånestad, Luca Guardabassi, Bart Pardon

**Affiliations:** 1 Infection Medicine, The University of Edinburgh, Edinburgh, UK; 2 International Veterinary Evidence-based Guidelines Centre, University of Copenhagen, Fredriksberg C, Denmark; 3 Veterinary Public Health Institute, University of Bern, Bern, Switzerland; 4 Department of Internal Medicine, Burdur Mehmet Akif Ersoy University, Burdur, Burdur Province, Turkey; 5 Scientific directorate of infectious diseases in animals, Sciensano, Brussels, Belgium; 6 Département des sciences cliniques, University of Montreal Faculty of Veterinary Medicine, Saint-Hyacinthe, Québec, Canada; 7 Centre for Veterinary Public Health & One Health, University of Veterinary Medicine Vienna, Vienna, Austria; 8 Department of Production Animal Clinical Sciences, Norwegian University of Life Sciences, Ås, Norway; 9 Comparative Biomedical Sciences, The Royal Veterinary College, London, England, UK; 10 Department of Pathobiology and Population Medicine, College of Veterinary Medicine, Mississippi State University, Mississippi, USA; 11 Norwegian Veterinary Institute, Ås, Norway; 12 Department of Veterinary and Animal Sciences, University of Copenhagen, Fredriksberg C, Denmark; 13 UGent Calf Health, Department of Internal Medicine, Reproduction and Population Medicine, Ghent University, Salisburylaan, Belgium

**Keywords:** antimicrobial stewardship- pneumonia- calves- inflammation- animal welfare

## Abstract

**Background:**

Non-steroidal anti-inflammatory drugs (NSAIDs) are widely used as ancillary therapy for bovine respiratory disease (BRD) alongside antimicrobials, and interest in NSAID monotherapy has grown amid antimicrobial stewardship and welfare concerns. We evaluated the clinical effectiveness of NSAIDs for BRD as adjunctive or sole therapy.

**Methods:**

We conducted a systematic review and meta-analysis of randomized controlled trials (RCTs) in cattle with naturally occurring BRD. Databases searched were MEDLINE (Ovid), Embase (Ovid), CAB Abstracts (Ovid), Biological Abstracts (Ovid), Web of Science Core Collection, and Scopus (search: 22 Apr 2024; update: 12 Sep 2025). Primary outcomes were short-term treatment failure (need for re-treatment); secondary outcomes included relapse, mortality, adverse events, and performance where available. Random-effects pairwise meta-analyses estimated risk ratios (RRs) with 95% CIs. Risk of bias was assessed with a modified RoB 2.0 tool; certainty of evidence was appraised using GRADE. Registration/protocol: this review extends a prior ENOVAT BRD protocol; no separate registration was created. Funding: COST Action CA18217.

**Results:**

Seventeen RCTs (22 comparisons; 4,909 animals) compared NSAID+antimicrobial versus antimicrobial alone. Adding an NSAID did not reduce re-treatment (RR 0.94, 95% CI 0.84–1.05; I ^2^ = 0%; moderate certainty). Subgroup (same vs different antimicrobial) and sensitivity analyses (handling of multi-arm trials; follow-up restricted to ≤14 or ≤ 10 days; risk-of-bias restrictions) did not change conclusions. Two RCTs in UK dairy calves compared NSAID monotherapy with antimicrobial monotherapy (RR 1.19, 95% CI 0.72–1.97; very low certainty). Re-treatment is an indirect outcome and may not capture analgesia, fever reduction, or growth effects.

**Conclusions:**

Across RCTs, NSAID use did not confer a clinically relevant reduction in re-treatment when added to antimicrobials, and evidence is very uncertain for NSAID monotherapy versus antimicrobials. Future trials should include validated pain/welfare measures, clinical and ultrasonographic outcomes, and performance metrics, with pathogen identification to explore effect modification.

## Introduction

Respiratory tract infections (RTIs) in cattle, commonly referred to as bovine respiratory disease (BRD), represent one of the most important health challenges in cattle production worldwide. BRD is a major cause of economic loss, compromised animal welfare and remains the leading indication for antimicrobial use in calves, youngstock and beef production ( Pardon et al., 2013, Bokma et al., 2018, Smith et al., 2020). Pneumonia in calves has been shown to be a painful condition and, when chronic, can result in prolonged suffering and impaired welfare ( Martin et al., 2022). Persistent public concern regarding intensive antimicrobial use in livestock has increased pressure on the sector to strengthen antimicrobial stewardship and to explore alternative or complementary strategies, with a particular focus on preventative approaches. Simultaneously, societal expectations regarding animal welfare increasingly influence the social license to operate of livestock production systems.

For decades, non-steroidal anti-inflammatory drugs (NSAIDs) have been used as adjunctive therapy to antimicrobials in the treatment of BRD. NSAIDs exert four principal effects: anti-inflammatory, antipyretic, anti-oedematous, and analgesic actions, although the relative expression of these effects varies among substances. In the context of BRD, the antipyretic and anti-inflammatory effects, particularly within the pulmonary tissue, have traditionally been considered the most relevant. The concomitant use of NSAIDs with antimicrobials has become standard practice in BRD therapy, culminating in the successful marketing of combination products containing both an antimicrobial and an NSAID from approximately 2006 onwards, with additional combinations introduced more recently. Conventional treatment paradigms are being challenged. Thoracic ultrasonography shows a higher prevalence of lung pathology than expected and there is mounting societal pressure to reduce antimicrobial use ( Jourquin et al., 2023). The continued importance of prudent antimicrobial use has increased interest in the use of NSAIDs as sole therapy in presumed viral or upper respiratory tract infections, and as primary treatment in mild pneumonia cases with the aim of preventing disease progression and avoiding antimicrobial administration ( Bassetti et al., 2024; Mahendran, 2020). Nevertheless, concerns have also been raised regarding NSAID use in RTIs. In human medicine, outpatient NSAID administration has been associated with prolonged hospitalization and extended duration of antimicrobial therapy ( Voiriot et al., 2019). Moreover, the clinical effects of NSAIDs may depend on the causative pathogen(s) and host status, with evidence suggesting neutral or even detrimental outcomes in certain contexts, including reports of non-steroidal anti-inflammatory drug–exacerbated disease in humans (NERD) ( Njoto et al., 2025). To the authors knowledge no studies have explored the existence of such effects in cattle.

To enable sustainable and evidence-based decision-making within the cattle industry, a comprehensive understanding of the effects of NSAID use on clinical cure, economic outcomes, and animal pain and welfare is required. To the authors’ knowledge, no meta-analysis on this topic is currently available. The only existing systematic review dates back to 2012 and was limited to direct comparisons using the same antimicrobial, resulting in the inclusion of only six studies ( Francoz et al., 2012).

Therefore, the primary aim of this systematic review and meta-analysis was to assess the clinical effectiveness of NSAID treatment for BRD either in combination with antimicrobials or as sole therapy. The results will inform the BRD Antimicrobial Use Guidelines of the European Network for Optimization of Antimicrobial Therapy (ENOVAT) and European Society of Clinical Microbiology and Infectious Diseases (ESCMID) Study Group for Veterinary Microbiology (ESGVM). The ENOVAT guidelines initiative encourages policymakers to use the results of this study in the development of regional or national antimicrobial treatment guidelines for BRD.

## Materials and methods

### Population, intervention, comparator and outcome (PICO) generation

The work presented in this paper stems from the development of antimicrobial treatment guidelines for BRD, a task undertaken by ENOVAT ( Damborg et al., 2024) and adhere to PRISMA reporting standards ( Page et al., 2021). The PRISMA checklist is reported in Supplementary File 1. A total of 10 PICO questions were co-created between panellists, farmers and clinicians during stakeholder interviews aimed at defining relevant review questions ( Scahill et al., 2026). This review extends the ENOVAT BRD antimicrobial treatment review protocol ( https://syreaf.org/wp-content/uploads/2022/07/BRD-PRISMA-protocol_version-for-upload_2022-07.pdf). No separate registration was created for the NSAID extension. The two PICO questions presented here were added *post hoc* during a ENOVAT panel meeting at the University of Copenhagen (March 2024), when the antimicrobial treatment recommendations were drafted. Experts and stakeholders felt the need for include recommendations regarding the use of NSAIDs within the context of BRD. The same clinical thresholds were used for the assessment of a clinically relevant effect also for the present PICOs ( Scahill et al., 2026). A trivial effect was considered to be: <200 treatment failures per 1000 animals; a small effect: 200–250 treatment failures; a moderate effect: 250–300 treatment failures; and a large effect >300 treatment failures.

PICO 1: Does the addition of NSAIDs contribute to more favourable outcomes in the treatment of bovine respiratory disease?

Population: Cattle with bovine respiratory disease.

Intervention: Antimicrobials (any) and NSAID (any).

Comparator: Antimicrobials.

Outcome(s): Short-term treatment failure (i.e., need for re-treatment), Relapse, Mortality, Adverse events.

PICO 2: Is NSAID treatment as efficacious as antimicrobials in the treatment of bovine respiratory disease?

Population: Cattle with bovine respiratory disease.

Intervention: NSAIDs (any).

Comparator: Antimicrobials (any).

Outcome(s): Short-term treatment failure (i.e., need for re-treatment), Relapse, Mortality.

### Sub-analysis

In PICO 1 (NSAID + ATB vs ATB alone), we included all antimicrobial regimens used in the intervention and comparator arms. To examine potential effect modification by the antimicrobial regimen, we conducted two subgroup analyses: (1) trials using the same antimicrobial in both arms (e.g., florfenicol + NSAID vs florfenicol alone) and (2) trials using different antimicrobials across arms (e.g., florfenicol + NSAID vs tulathromycin). In addition, because most trials administered flunixin meglumine, we performed a *post hoc* subgroup analysis by NSAID agent to assess whether flunixin meglumine specifically modified the effect.

### Search strategy and eligibility criteria

MEDLINE, Embase, CAB Abstracts, Biological Abstracts, Web of Science, and Scopus databases were searched on 2024-04-22. The search was updated on 2025-09-12. Embase was unavailable for the 2025 update. A senior librarian performed the search and was consulted in the search strategy. The search string and search report can be found in Supplementary file 2. Peer reviewed controlled trials of cattle with natural infection and clinical signs of BRD were eligible for inclusion. Cattle of all ages and production systems, irrespective of bacterial aetiology, were included as well as trials with unknown bacterial agents. Other co-treatments, including metaphylaxis prior to trial enrolment, were accepted if both intervention and comparator groups received the treatment. Trials that evaluated solely the effect of metaphylaxis, articles written in languages other than English, and conference proceedings were excluded. Grey literature was not searched.

### Study selection and data collection

Four reviewers (MC, RY, KS, LPC) independently screened titles, abstracts, and full texts in duplicate using the Covidence platform ( www.covidence.org). Calibration exercises were undertaken at each stage of study selection and data extraction to refine procedures and promote standardization across reviewers. Data extraction was also conducted independently in duplicate (KS, RY, MC, LPC) and captured details on the study population and baseline characteristics (age, production system), assessment methods, microbiological sampling (methods, isolated bacteria, presence of Mycoplasma, *in vitro* antimicrobial resistance), interventions and comparators (substance, dosage, frequency, duration), and potential conflicts of interest and funding. When outcomes were reported only as percentages and group totals were available, we back-calculated event counts by multiplying the percentage by the group total and rounding to the nearest integer. All data were entered into a data management tool (Excel, Microsoft). Any disagreements between reviewers were resolved through discussion.

### Data synthesis and sensitivity analysis

We performed a direct pairwise meta-analysis using a random-effects model to estimate the pooled risk ratio across studies. Cochrane’s Review Manager ( revman.cochrane.org) was used to generate the meta-analyses. Effect size was presented in absolute risk difference and risk ratio with a 95% confidence interval (CI). Descriptive summaries were provided for data that could not be pooled. Some trials were multi-arm with one study group shared across more than one eligible comparison (e.g., multiple NSAID+antimicrobial arms sharing a single antimicrobial-only comparator, or one NSAID+antimicrobial arm compared with two different antimicrobial comparators). In the primary analysis, we retained by-molecule contrasts but avoided double-counting by splitting the shared group approximately equally across comparisons, dividing both events and totals so that split counts summed to the original. Due to the heterogenity of study duration (range 3 days to 90 days) we conducted two outcome-window sensitivity analyses: (i) restricting follow-up to ≤14 days by excluding trials that reported outcomes beyond 14 days or did not report the follow-up duration (NR); and (ii) a more conservative restriction to ≤10 days, excluding trials with outcomes reported after 10 days or with unreported follow-up (NR). We also conducted a sensitivity analysis restricted to trials at low risk of bias across all RoB domains (modified RoB 2.0), excluding trials with some concerns or high risk.

### Certainty assessment

Certainty of evidence was appraised by KS using the GRADE approach that consists of five domains: risk of bias, indirectness, inconsistency, imprecision and publication bias ( Guyatt et al., 2025), with the panel and methodology task force contributing to discussions and final decisions on the certainty ratings. Inconsistency (heterogeneity) among included studies was assessed primarily by visual inspection to identify outliers relative to predefined clinical thresholds, rather than relying on I ^2^ statistics, in line with GRADE guidance ( Guyatt et al., 2023). Imprecision was evaluated using the confidence-interval approach; effect estimates were considered precise if the 95% CI did not cross any clinical thresholds ( Zeng et al., 2022). GRADEpro ( www.gradepro.org) was used to produce summary of findings (SoF) tables and to calculate risk differences for anticipated absolute effects. Risk of bias was assessed using the Cochrane RoB 2 tool ( Sterne et al., 2019), operationalised with signalling questions adapted for livestock trials ( Moura et al., 2019). Two reviewers (KS, LPC), worked independently in duplicate and any disagreements were resolved by discussion. Publication bias was assessed with a funnel plot for PICO 1 by using the Cochrane’s Review Manager ( revman.cochrane.org).

## Results

### PICO 1 – Does the addition of NSAIDs contribute to more favourable outcomes in the treatment of bovine respiratory disease?

**Included studies**

**Figure 1.**
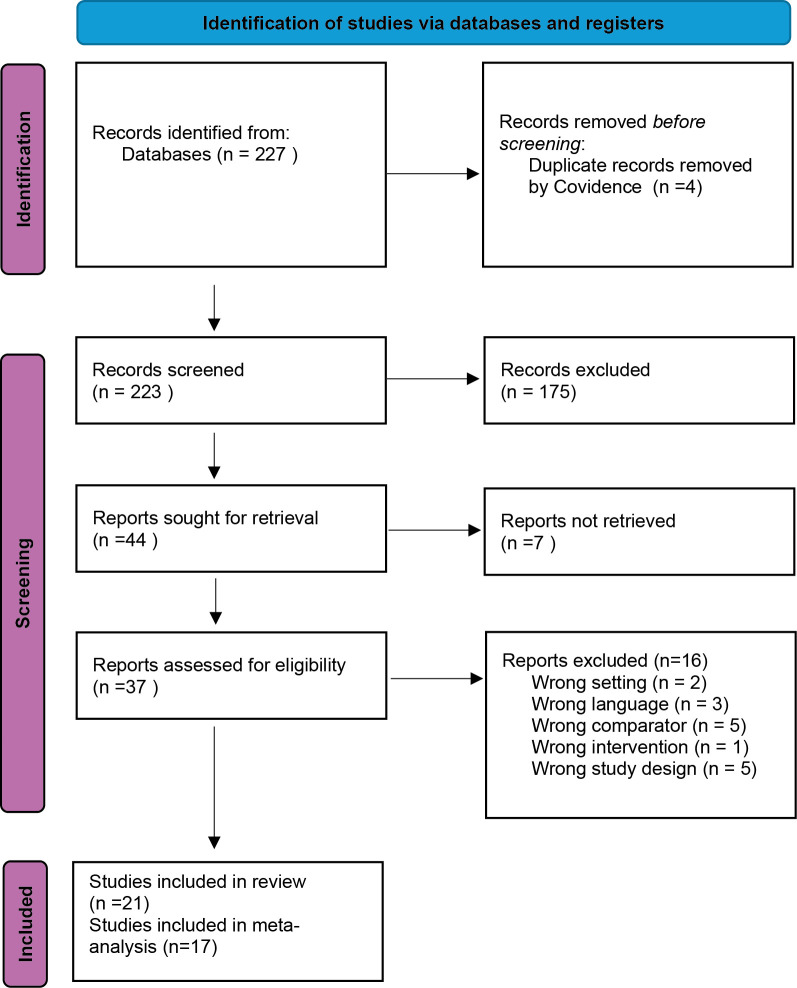
PRISMA 2020 flow diagram for study selection (PICO 1: NSAID + antimicrobial vs antimicrobial alone). Database searches were run on 22 Apr 2024 (MEDLINE [Ovid], Embase [Ovid], CAB Abstracts [Ovid], Biological Abstracts [Ovid], Web of Science Core Collection, Scopus) and updated on 12 Sep 2025 (MEDLINE, CAB Abstracts, Biological Abstracts, Web of Science, Scopus; Embase unavailable for the update). Twenty-one studies were included in the review, of which 17 contributed to the meta-analysis.

PICO 1: Twenty-one randomised controlled trials (RCT) met the inclusion criteria and compared NSAID and antimicrobials with antimicrobials alone. The number of excluded reports and reasons for exclusion for retrieved texts are reported in Figure 1. Absolute numbers (animals and number of treatment failures) could not be extracted from four trials and they were thus not included in the meta-analysis ( Bednarek et al., 2003, Bednarek et al., 2013, Elitok and Elitok, 2004, Nickell et al., 2020). Authors of these four trials were contacted for information about absolute numbers but no responses were received; the results for these four trials are therefore reported descriptively in Supplementary File 3. Seventeen trials and twenty-two arms including a total of 4909 animals could be pooled in a meta-analysis ( Deleforge et al., 1994; Scott, 1994; Lockwood et al., 2003; Friton et al., 2005; Keita et al., 2007; Hannon et al., 2009; Van Donkersgoed et al., 2009; Guzel et al., 2010; Thiry et al., 2014; Van Donkersgoed et al., 2014; Behlke et al., 2015; Mahendran, 2020; Bringhenti et al., 2021; De Koster et al., 2022; Pollreisz et al., 2022; Ferree et al., 2023; Tomazi et al., 2023). Most pooled trials were from North America (n = 9) or Europe (n = 6), and there was one trial from Turkey in Eurasia and one trial from Mexico in Central America. Median study duration was 10 days (range 3–90 days). Study characteristics for all pooled trials such as country, production system, age, antimicrobial and NSAID substances including dosage, duration of treatment, study duration and *Mycoplasmopsis bovis s*tatus are reported in Table 1. The full data extraction for all articles are presented in Supplementary File 3.

**Table 1.** Study characteristics for all included studies in PICO 1 and PICO 2.

PICO	First author (year)	Country	Production system	Age	Intervention	Comparator	Duration of treatment	Study duration ^a^ (days)	Mycoplasma positive animals
1	Deleforge et al. (1994)	France, Belgium	Not reported	Not reported	Oxytetracycline (20 mg/kg, im) and tolfenamic acid (2 mg/kg)	Oxytetracycline (20 mg/kg, im.)	One or two injections (Oxytetracycline after 72 h and tolfenamic acid after 48 h)	21	Unknown. No sampling
1	Bringhenti et al. (2021)	USA	Dairy	Preweaned	Florfenicol (40 mg/kg, sc) and flunixin meglumine (2,2 mg/kg sc)	Tildipirosin (4 mg/kg)	One injection. New treatment allowed after 5 days if no respons	5	Yes
1	De Koster et al. (2022)	Germany, Portugal	Beef and dairy	Not reported	Tulathromycin (2.5 mg/kg, sc) and ketoprofen (3 mg/kg, sc)	Tulathromycin (2.5 mg/kg, sc) and placebo	One injection	14	Yes
1	Ferree et al. (2023)	USA	Dairy	Preweaned	Tulathromycin (1.1 mL/kg, SC, once) and meloxicam (1 mg/kg, po)	Tulathromycin (1.1 mL/kg, SC, once) and placebo	One injection	7	Unknown. No sampling
1	Friton et al. (2005)	Mexico	Feedlot	Not reported	Oxytetracycline (20 mg/kg, sc) and meloxicam (0.5 mg/kg. sc)	Oxytetracycline (20 mg/kg, sc) and saline	One injection	7	Unknown. Not sampled
1	Keita et al. (2007)	France	Slaughter	8–90 days	Oxytetracycline (20 mg/kg, i.m.) and flunixin meglumine	Oxytetracycline (30 mg/kg, i.m.)	One injection	10	Yes. Cultured on 2 farms and positive seroconversion on 3 farms
1	Guzel et al. (2010)	Turkey	Not reported	Not reported	Tulathromycin (2.5 mg/kg, sc) and flunixin meglumine (2.2 mg/kg, sc) OR diclofenac sodium (2.5 mg/kg, sc)	Tulathromycin (2.5 mg/kg, sc)	One injection	7	Unknown. No sampling
1	Lockwood et al. (2003)	USA	Beef	Not reported	Ceftiofur (1.1 mg/kg im.) and flunixin meglumine (2.2 mg/kg iv) OR ketoprofen (3 mg/kg) OR carprofen (1.4 mg/kg)	Ceftiofur (1.1 mg/kg im.)	Ceftiofur for 3 days, NSAIDs given once	14	Unknown. No sampling
1	Mahendran (2020)	United Kingdom	Dairy	<10 weeks	Florfenicol (40 mg/kg, sc) and flunixin meglumine (3.3 mg/kg sc)	Florfenicol (40 mg/kg, sc)	NSAID given 3 days, antimicrobial one injection	10	No exposure according to serology of five calves
1	Scott (1994)	United Kingdom	Beef	2–5 months	Tilmicosin (10 mg/kg, sc) and flunixin meglumine (2.2 mg/kg, iv)	Tilmicosin (10 mg/kg, sc)	One injection	Not reported	Serology negative
1	Thiry et al. (2014)	Belgium, France, Spain	Veal	Pre-ruminating	Florfenicol (40 mg/kg, sc) and flunixin meglumine (2,2 mg/kg sc)	Florfenicol (40 mg/kg, sc)	One injection	10	Yes
1	Tomazi et al. (2023)	USA	Dairy	Preweaned	Florfenicol (40 mg/kg, sc) and flunixin meglumine (2,2 mg/kg sc)	Tildipirosin (4 mg/kg, sc)	One injection	10	Yes
1	Pollreisz et al. (2022)	USA	Feedlot	Not reported	Florfenicol (40 mg/kg, sc) and flunixin meglumine (2,2 mg/kg sc)	Ceftiofur (6.6 mg/kg im.)	One injection	90	Unknown. No sampling
1	Van Donkersgoed et al. (2014)	Canada	Feedlot	6–10 months	Florfenicol (40 mg/kg, sc) and flunixin meglumine (2,2 mg/kg sc)	Florfenicol (40 mg/kg, sc) OR Tilmicosin (10 mg/kg, sc)	One injection. New treatment allowed after 5 days if no respons	Not reported	Unknown. No sampling
1	Behlke et al. (2015)	Canada	Feedlot	Not reported	Florfenicol (40 mg/kg, sc) and flunixin meglumine (2,2 mg/kg sc)	Ceftiofur (6.6 mg/kg sc.)	One injection	Until slaughter	Unknown. No sampling
1	Hannon et al. (2009)	Canada	Feedlot	Not reported	Florfenicol (40 mg/kg, sc) and flunixin meglumine (2 mg/kg sc)	Ceftiofur (6.6 mg/kg sc.) or tulathromycin 2.5 mg/kg SC (multi-arm)	One injection	Until harvest	
1	Van Donkersgoed et al. (2009)	Canada	Feedlot	Not reported	Florfenicol (40 mg/kg, sc) and flunixin meglumine (2,2 mg/kg sc)	Tulathromycin (2.5 mg/kg, sc)	One injection	Not reported	Unknown. No sampling
2	Mahendran et al. (2017)	United Kingdom	Dairy	<10 weeks	Flunixin meglumine (2 mg/kg sc. SID)	Gamithromycin (6 mg/kg.im)	NSAID given 3 days, antimicrobial one injection	10	Unknown. Not sampled
2 (also in PICO 1 above)	Mahendran (2020)	United Kingdom	Dairy	<10 weeks	Flunixin meglumine (3.3 mg/kg sc)	Florfenicol (40 mg/kg, sc)	NSAID given 3 days, antimicrobial one injection	10	No exposure according to serology of five calves

^a^ Days when pooled outcome was measured. i. m. = intramuscular, s.c. = subcutaneous, SID = once a day, EOD = every other day.

**Treatment efficacy**

**Figure 2.**
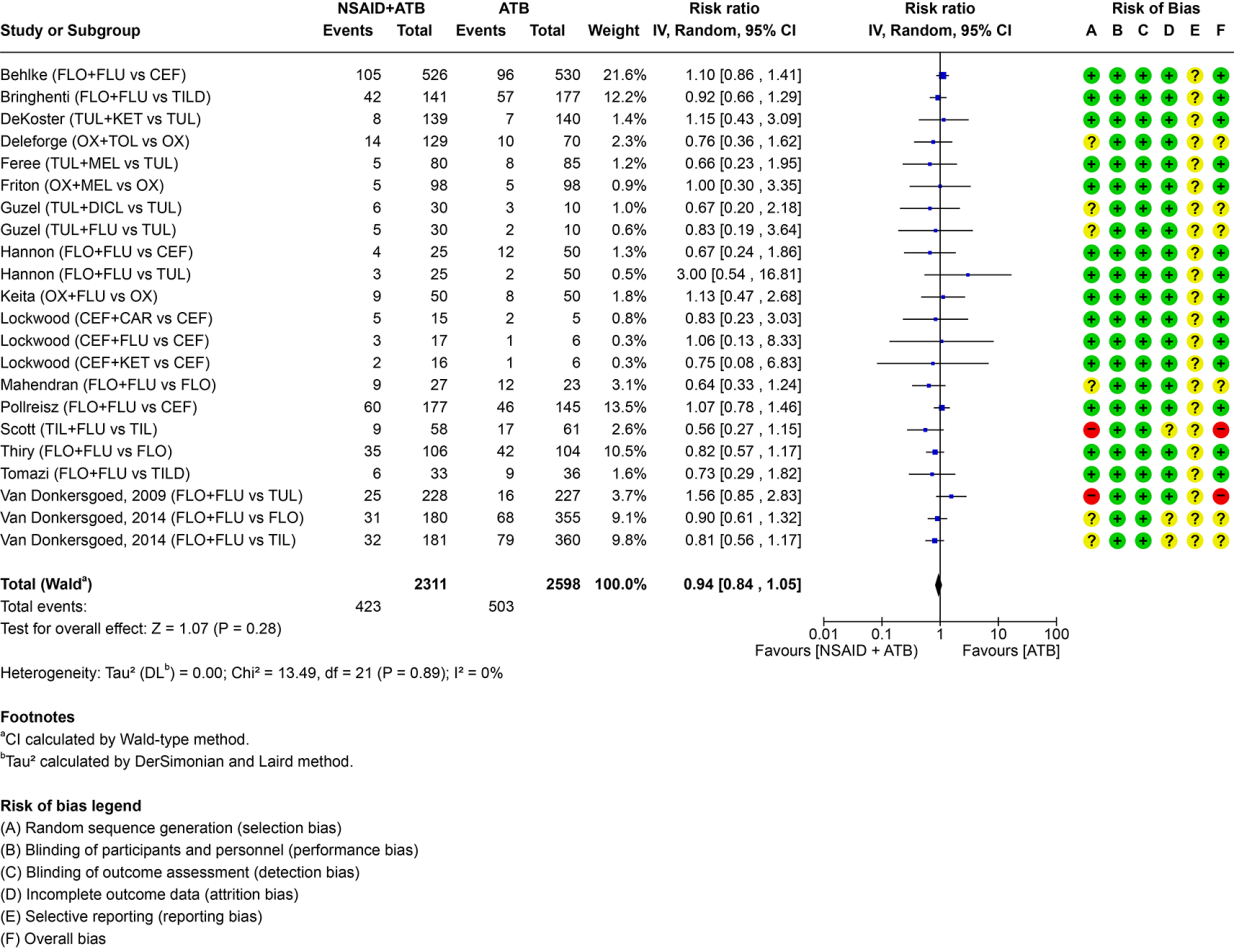
Forest plot for PICO 1 comparing the efficacy of non-steroidal anti-inflammatory drugs (NSAIDs) given with an antimicrobial (ATB) versus antimicrobial alone. The number of events represents the need for re-treatment. For multi-arm trials with a shared study arm, the shared group was split equally across comparisons (events and totals) to avoid double counting. In the risk of bias assessment a red “traffic light” means high risk of bias, yellow means that there are some concerns and green that the risk of bias is low. FLO = florfenicol; CEF = ceftiofur; OX = oxytetracycline; TUL = tulathromycin; TIL = tilmicosin; TILD = tildipirosin. FLU = flunixin meglumine; KET = ketoprofen; CAR = carprofen; MEL = meloxicam; TOL = tolfenamic acid; DICL = diclofenac (sodium).

The addition of NSAID did not result in a clinically relevant difference in the number of re-treatments in comparison to antimicrobials only in cattle with BRD. The pairwise comparisons including individual study results are visualized in the forest plot ( Figure 2). Absolute risk difference, risk ratio, *p*-value and overall certainty of evidence is reported in Table 2 and summary of findings tables can be found in Supplementary File 4.

**Table 2.** Efficacy results for all pooled PICOs shown in absolute numbers, p-values, risk ratio with 95% confidence intervals (CI), and certainty of evidence.

PICO	Absolute risk difference	*p*-value	Risk ratio (95% CI)	Certainty of evidence
1	NSAID and antimicrobial treatment resulted in 12 fewer therapeutic failures per 1000 animals (from 31 fewer to 10 more) in comparison to antimicrobials alone.	0.28	0.94 (0.84–1.05)	Moderate
2	NSAID treatment resulted in 88 more therapeutic failures per 1000 animals (from 129 fewer to 448 more) in comparison to antimicrobials.	0.50	1.19 (0.72–1.97)	Very low

**Certainty of evidence**

**Figure 3.**
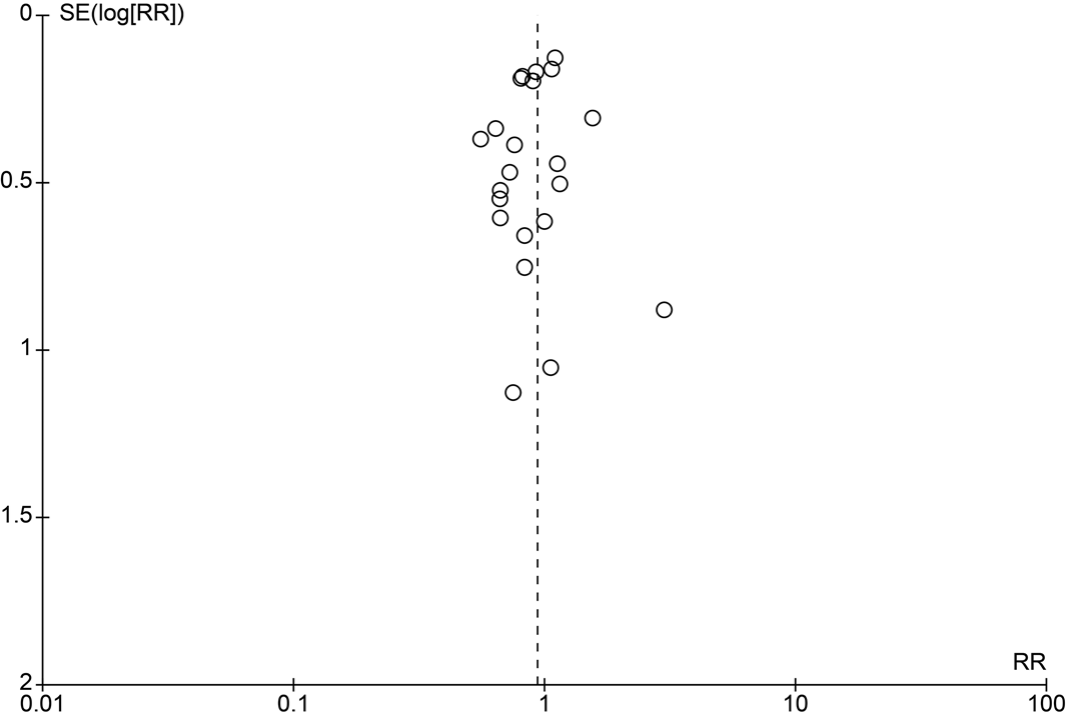
Funnel plot for PICO 1 (NSAID + antimicrobial vs antimicrobial alone). Each circle represents one study (one comparison per study; multi-arm trials were combined to avoid double counting). The x-axis shows the risk ratio (RR) on a logarithmic scale; the y-axis shows the standard error of the log RR [SE (log RR)] with larger studies at the top. The dashed vertical line indicates the line of no effect (RR = 1). Visual inspection suggests an approximately symmetric distribution, providing no clear evidence of small-study effects or publication bias; however, interpretation is limited by the modest number of studies and clinical/analytical heterogeneity inherent to the dataset.

The overall certainty of evidence (CoE) was moderate. The only domain that was downgraded was the indirectness domain ( Table 3) due to indirectness of the reported outcome. The outcome reported was the need for re-treatment, which is considered a reasonable surrogate outcome for overall health, as animals not requiring re-treatment are unlikely to have overt clinical signs. However, the panelists were concerned that this did not fully capture the full indication for NSAID treatment, particularly its role in alleviating pain. Speed of recovery (e.g. reducing pyrexia) was investigated in some of the studies, but this outcome could not be pooled and was therefore only reported descriptively in Supplementary File 3. Two studies were at high risk of bias and four had some concerns. Effect estimates from these studies were consistent with those from low risk-of-bias trials and remained within our predefined threshold for a clinically relevant effect and we therefore did not rate down for risk of bias. Risk-of-bias judgements by domain are shown in Figure 2, with full assessments in Supplementary File 5. Sensitivity analyses excluding high risk-of-bias trials and restricting to low risk-of-bias trials produced estimates similar to the primary analysis (RR 0.99 [95% CI 0.86–1.14]) (Supplementary File 6). Publication bias was assessed by a funnel plot ( Figure 3), which was symmetrical and showed no obvious reasons for concern.

**Table 3.** Certainty assessment for all domains and the overall certainty for the therapeutic failure for PICO 1 and 2.

PICO	Risk of bias	Indirectness	Inconsistency	Imprecision	Publication bias	Overall certainty
1	Not serious	Serious	Not serious	Not serious	Undetected	Moderate
2	Serious	Serious	Not serious	Extremely serious	Undetected	Very low

**Sub-analysis**

None of the sub-analyses showed an effect modification of clinical relevance. The absolute risk difference was slightly larger (35 fewer therapeutic failures per 1000 animals) in the trials comparing the same antimicrobials, than in the trials that compared different antimicrobials (4 fewer therapeutic failures per 1000 animals). However, the confidence intervals (ranging from 60 fewer to 2 fewer) for the analysis only including the same antimicrobial comparators were well within the range of a clinically trivial effect of 200 therapeutic failures per 1000 animals. The pooled results of the sub-analysis of flunixin meglumine alone was almost identical to the pooled results of all other NSAIDs. Absolute risk difference, risk ratio, p-value and overall certainty of evidence for all sub-analyses are reported in Table 4. Forest plots for all sub-analysis can be seen in Supplementary File 6.

**Table 4.** Efficacy results for sub-analysis shown in absolute numbers, p-values, risk ratio with 95% confidence intervals (CI), and certainty of evidence.

PICO	Subanalysis	Absolute effect	*p*-value	Risk ratio (95% CI)	Certainty of evidence
1	Same antimicrobial comparator	NSAID and antimicrobial treatment resulted in 35 fewer therapeutic failures per 1000 animals (from 60 fewer to 2 fewer) in comparison to antimicrobials alone.	0.04	0.81 (0.67–0.99)	Moderate
1	Different antimicrobial comparator	NSAID and antimicrobial treatment resulted in 4 fewer therapeutic failures per 1000 animals (from 24 fewer to 34 more) in comparison to antimicrobials alone.	0.83	1.02 (0.88–1.17)	Moderate
1	Flunixin meglumine only	Flunixin meglumine and antimicrobial treatment resulted in 11 fewer therapeutic failures per 1000 animals (from 34 fewer to 15 more) in comparison to antimicrobials alone.	0.41	0.95 (0.84–1.07)	Moderate
1	Flunixin meglumine excluded	NSAID and antimicrobial treatment resulted in 16 fewer therapeutic failures per 1000 animals (from 40 fewer to 21 more) in comparison to antimicrobials alone.	0.35	0.82 (0.54–1.24)	Moderate

**Sensitivity analysis**

Several included trials were multi-arm ( Guzel et al., 2010; Hannon et al., 2009; Lockwood et al., 2003; Van Donkersgoed et al., 2014) we corrected unit-of-analysis error in the primary meta-analysis by splitting shared groups across relevant comparisons (see Methods). The pooled effect was RR 0.94 (95% CI 0.84–1.05; I ^2^ = 0%). In a sensitivity analysis where multiple eligible arms within each multi-arm trial were combined into a single comparison, the pooled effect was unchanged (RR 0.94, 95% CI 0.83–1.05; I ^2^ = 0%), confirming robustness (forest plot for sensistivity analysis is presented in Supplementary File 6). In a sensitivity analysis limited to trials reporting outcomes within 14 days (excluding NR), the pooled effect was RR 0.85 (95% CI 0.70–1.03; I ^2^ = 0%). In a more conservative analysis restricted to ≤10 days, the pooled effect was RR 0.84 (95% CI 0.69–1.03; I ^2^ = 0%). Both estimates remained below our predefined threshold for a clinically relevant effect and did not change the conclusions of the primary analysis. Forest plots are provided in Supplementary File 6 (Figures S7 and S8).

### PICO 2 – Is NSAID treatment as effective as antimicrobials in the treatment of bovine respiratory disease?

**Included studies**

**Figure 4.**
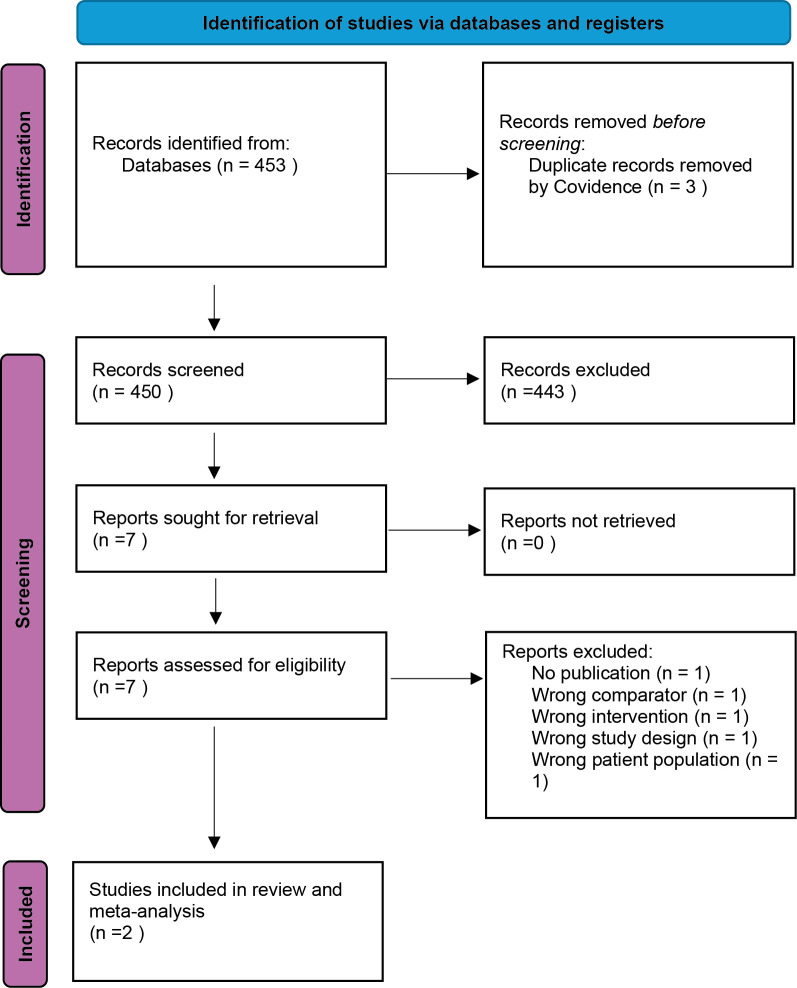
PRISMA 2020 flow diagram for study selection (PICO 2: NSAID monotherapy vs antimicrobial monotherapy). Database searches were run on 22 Apr 2024 in MEDLINE (Ovid), Embase (Ovid), CAB Abstracts (Ovid), Biological Abstracts (Ovid), Web of Science Core Collection, and Scopus, and updated on 12 Sep 2025 in MEDLINE, CAB Abstracts, Biological Abstracts, Web of Science, and Scopus (Embase unavailable for the update). Two studies met inclusion criteria and were included in the review and meta-analysis.

Two trials compared NSAID monotherapy with antimicrobial monotherapy ( Mahendran et al., 2017; Mahendran, 2020). The number of excluded reports and reasons for exclusion for retrieved texts are reported in Figure 4. Both studies were conducted in the United Kingdom. Study characteristics of the selected studies can be seen in Table 1 and the full data extraction can be seen in Supplementary file 3.

**Treatment efficacy and certainty of evidence**

**Figure 5.**
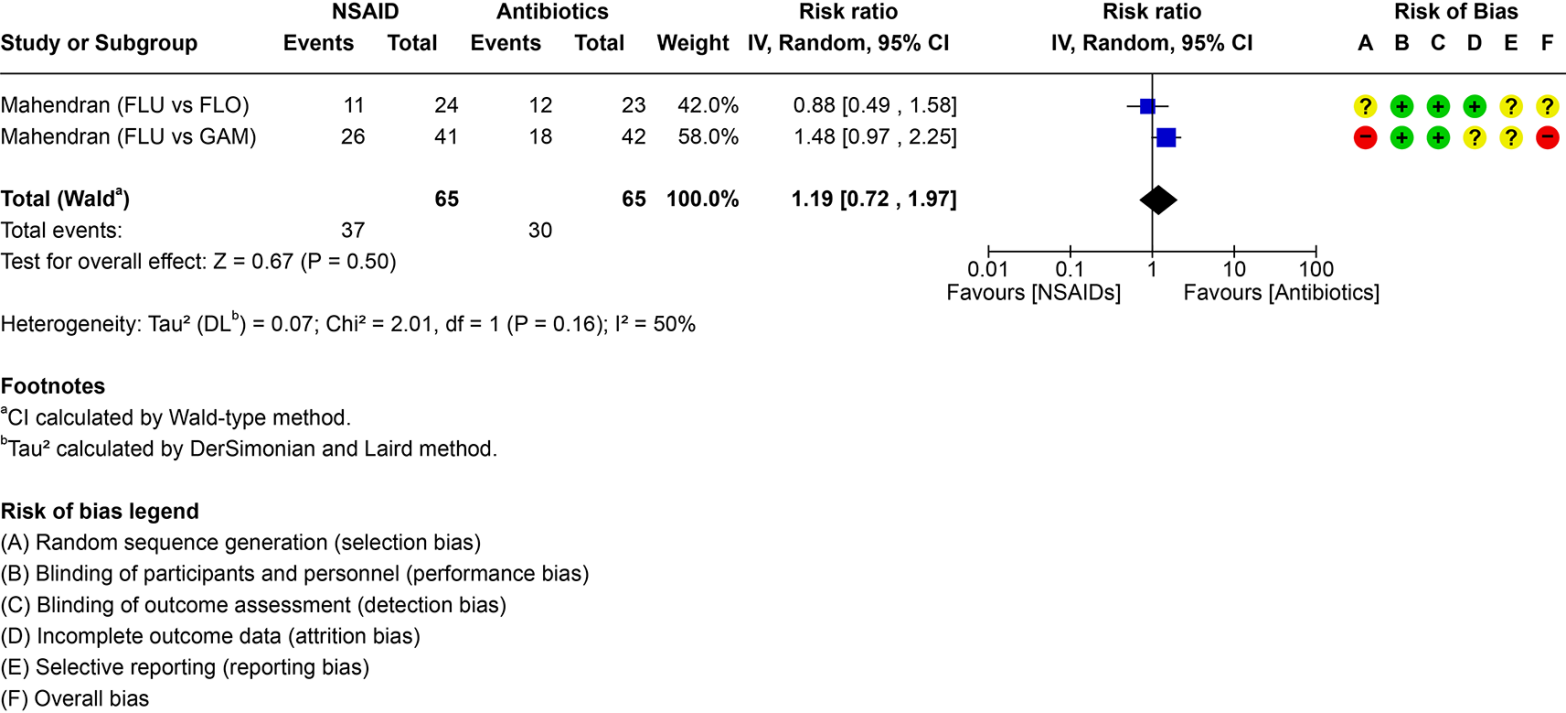
Forest plot for PICO 2 comparing NSAID monotherapy versus antimicrobial (ATB) monotherapy. The number of events represents the need for re-treatment. In the risk of bias assessment, a red “traffic light” means high risk of bias, yellow means that there are some concerns and green that the risk of bias is low. FLU = flunixin meglumine, FLO = florfenicol; GAM = gamithromycin.

Absolute risk difference was not clinically relevant but the precision was extremely uncertain and ranged from 129 fewer to 448 more per 1000 animals which would be considered a large effect (>200 animals) of animals experiencing treatment failure ( Table 2). The pairwise comparisons including individual study results are visualized in the forest plot ( Figure 5). The imprecision domain was rated down three levels because three clinical thresholds were crossed, resulting in an overall certainty of evidence rated as very low ( Table 3).

## Discussion

The main finding (PICO 1) of the present meta-analysis was that moderate certainty evidence from seventeen randomised controlled trials, including 4909 cattle, showed that adding NSAIDs to an antimicrobial treatment regimen did not result in a clinically beneficial effect with respect to the need for re-treatment. Moderate certainty evidence means that the *true effect is probably close to the estimated effect* ( Guyatt et al., 2025). In order to correctly frame these results, important limitations must be mentioned. The present study used the outcome of “need for re-treatment”, as a measure of therapy failure. The studies varied in follow-up period duration (median duration 10 days) and follow-up intensity, ranging from 3 to 90 days, but a sensitivity analysis did not show a difference in effect size when only trials with a follow up period of ≤14 days or ≤ 10 days were included. Lung ultrasonography studies have made it undoubtedly clear that the prevalence of subclinical pneumonia is high and that clinical signs, albeit as a scoring system or as individual sign, are unable to reliably identify calves with pneumonia ( Lowie et al., 2022). Cure consists of three aspects which are time-dependent, namely bacteriological cure, clinical cure and pathological cure. Clinical cure typically precedes pathological cure ( Kuru & Lynch, 1999), potentially resulting in too early cessation of antimicrobial or anti-inflammatory therapy and subsequently clinical relapse from the same pathological lesion days to weeks later. Recently, lung reaeration and regression of lung consolidation were suggested as potentially more objective cure criteria, making it possible to differentiate different pneumonic episodes over time ( Jourquin et al., 2022, Jourquin et al., 2025a, Jourquin et al., 2025b). Bringing this back to the present meta-analysis, it cannot be excluded that NSAIDs had an effect on pathological cure but did not alter clinical cure. Similarly, for vaccines in the absence of effects on clinical signs, significant effects were seen on lung ultrasonographic findings ( Jourquin et al., 2023). Furthermore, for farmers, the economic return on investment, meaning effects on outcomes such as average daily liveweight gain or carcass weight, are predominant drivers in the decision making for NSAID use. These outcomes were not considered in the present meta-analysis (and were not documented in most studies). In conclusion, the “need for re-treatment” outcome did not consider production related benefits and animal welfare aspects, such as pain and speed of recovery (incl. Fever reduction), leading to a downgrade of the evidence for indirectness of the outcome. Otherwise, the results of this meta-analysis would have been of high certainty. The authors would, however, like to underscore that the analgesic effects and welfare benefits can be a very important reason to administer NSAIDs for BRD, and more research in this area should be a priority.

Considering the second PICO (2), the ability of monotherapy with NSAIDs to provide equal cure compared to antimicrobial therapy, available information was very limited, with only two studies, both conducted by the same author in a single country and restricted to one NSAID (flunixin). As mentioned, the interest in the use of NSAID as sole therapy in BRD has substantially increased, in such a way that recommendations have been made that NSAIDs should replace antimicrobials to a certain extent. In a first trial, calves were enrolled by means of automated fever tags (temperature threshold >39.7 °C) and received either flunixin meglumine for three days or the long acting antimicrobial, gamithromycin ( Mahendran et al., 2017). Flunixin-treated calves were five times more likely to require re-treatment within 72 hours. In a second trial, calves were also enrolled when fever tag temperature exceeded 39.7 °C, and a total of 24 calves were treated with flunixin meglumine and 23 calves with florfenicol ( Mahendran, 2020). Thoracic ultrasonography performed within 48 h of enrolment only showed the presence of comet tails, and no lung consolidation in the majority of cases. A trend towards a higher likelihood for repeat treatment was found in the NSAID only group ( Mahendran, 2020). While the second study can provide some information on the treatment of calves with upper respiratory tract infection that has not yet developed into pneumonia, the question of how beneficial NSAIDs are as sole therapy for pneumonia, especially mild pneumonia (lung consolidation <1 cm), remains unresearched. In the available trials the ethical inability to include true negative control groups in clinical cases makes it difficult to distinguish drug efficacy from spontaneous recovery.

The authors would like to state that the anti-inflammatory effect of NSAID is not necessarily beneficial for the inflammatory process caused by each pathogen. Indeed, a recent review for pneumonia in human medicine concluded that NSAIDs may reduce the odds of cure ( Voiriot et al., 2019). Also in cattle, potential negative effects of NSAIDs have been observed in experimental infections with bovine respiratory syncytial virus ( Hägglund et al., 2024). Whether this is the case for pneumonia in cattle urgently needs further exploration.

Interpretation of NSAID effects in BRD is complicated by bidirectional pharmacological interactions between NSAIDs and antimicrobials. Although NSAIDs lack clinically relevant antimicrobial activity, experimental studies have shown that some NSAIDs can modulate bacterial susceptibility to antimicrobials *in vitro*, including tetracyclines, via effects on membrane permeability and efflux ( Magnowska et al., 2019). At the same time, several antimicrobial classes commonly used for BRD, notably tetracyclines and macrolides, exert intrinsic anti-inflammatory or immunomodulatory effects independent of direct antibacterial effects ( Ruh et al., 2017). Overlapping pharmacodynamic effects may therefore obscure any incremental benefit of NSAID co-administration. Conversely, antimicrobials such as florfenicol, which lack well-established anti-inflammatory properties, might theoretically permit clearer assessment of NSAID add-on effects. However, available evidence, including subgroup analyses in the present study, does not support clinically relevant effect modification by antimicrobial class. Together, these observations highlight the complexity of NSAID–antimicrobial interactions and the difficulty of detecting additive effects in clinical trials.

In conclusion, either NSAIDs do not have an effect on the likelihood of cure in BRD, or the need for re-treatment based on clinical signs was not a good parameter to reliably evaluate their significance in BRD therapy. The available BRD literature has not directly evaluated pain relief, leaving the analgesic benefit of NSAIDs in BRD uncertain. Rather than implying lack of efficacy, this evidence gap supports re-evaluation of current labels and future studies using contemporary outcome measures (lung ultrasonography, validated pain/welfare scores, economic parameters such as growth performance, and pathogen identification).

## Ethics and consent

Not required. This article is a systematic review and meta-analysis of previously published studies and analyses only aggregate, study-level data available in the public domain (plus any aggregate numbers provided by corresponding authors upon request). No new experiments involving animals or humans were conducted by the authors, and no identifiable personal data were accessed. Consent to participate: Not applicable. Consent for publication: Not applicable.

## Data Availability

Open Science Framework (OSF): Supplementary files for manuscript: Efficacy of non-steroidal anti-inflammatory drug (NSAID) treatment for bovine respiratory disease: a systematic review and meta-analysis for the European Network for Optimization of Antimicrobial Therapy guidelines: https://doi.org/10.17605/OSF.IO/8SDMH ( Scahill, 2026). Supplementary File 3.xlxs (full data extraction; study/comparison/arm-level dataset including group totals, event counts for re-treatment time points used for pooling, and study characteristics/metadata used in analyses). Open Science Framework (OSF): Supplementary files for manuscript: Efficacy of non-steroidal anti-inflammatory drug (NSAID) treatment for bovine respiratory disease: a systematic review and meta-analysis for the European Network for Optimization of Antimicrobial Therapy guidelines: https://doi.org/10.17605/OSF.IO/8SDMH ( Scahill, 2026). This project contains the following extended data: Supplementary File 1.docx (PRISMA 2020 checklist). Supplementary File 2.docx (full search strategies and run reports). Supplementary File 4.docx (GRADE Summary-of-Findings tables). Supplementary File 5.xlxs (risk-of-bias forms and domain-level judgements). Supplementary File 6.docx (forest plots for subgroup and sensitivity analyses). Data are available under the terms of the Creative Commons Attribution 4.0 International license (CC-BY 4.0). The PRISMA 2020 checklist is provided in Supplementary File 1 and available at Open Science Framework https://doi.org/10.17605/OSF.IO/8SDMH ( Scahill, 2026). Data are available under the terms of the Creative Commons Attribution 4.0 International license (CC-BY 4.0).
