# Supplementary material for: Efficacy of non-steroidal anti-inflammatory drug (NSAID) treatment for bovine respiratory disease: a systematic review and meta-analysis for the European Network for Optimization of Antimicrobial Therapy guidelines
Source: Open Res Eur. 2026 May 4;6:77. Originally published 2026 Mar 18. [Version 2] doi: 10.12688/openreseurope.23174.2 (PMC13153766; doi:10.12688/openreseurope.23174.2)
Supplement: Peer review history

# Reviewer report: Reviewer response for version 2

**DOI:** 10.21956/openreseurope.25698.r73843

**Published:** 2026-05-27

**Authors:** Sarah M Raabis

**Affiliations:** 1 Colorado State University, Fort Collins, Colorado, USA

Overall, the meta-analysis is well-written and the topic is very relevant to bovine veterinary medicine. The analyses and limitations of the study design are well-described. 

 Minor comments:

 I think it would be helpful to discuss the distribution of beef and dairy populations within the studies that were included in the analyses within the discussion. I recognize that these populations are listed within Table 1, but I think it would provide more context for the interpretation. 

 Additionally, was there a reason that the analysis was limited to Mycoplasmopsis identification versus other bacterial or viral agents? It would be helpful to add this information to the discussion.

Are the rationale for, and objectives of, the Systematic Review clearly stated?

Yes

Is the statistical analysis and its interpretation appropriate?

Yes

Are sufficient details of the methods and analysis provided to allow replication by others?

Yes

Are the conclusions drawn adequately supported by the results presented in the review?

Yes

Reviewer Expertise:

I am a BRD researcher with experience in field trials, lung ultrasonography, nasopharyngeal and lung microbiome and transcriptome analyses.

I confirm that I have read this submission and believe that I have an appropriate level of expertise to confirm that it is of an acceptable scientific standard.

# Reviewer report: Reviewer response for version 2

**DOI:** 10.21956/openreseurope.25698.r73522

**Published:** 2026-05-07

**Authors:** Naod Thomas Masebo

**Affiliations:** 1 Bologna University, Bologna, Italy

I have accepted the explanation provided by the authors regarding the comment I suggested. 

 No further comment.

Are the rationale for, and objectives of, the Systematic Review clearly stated?

Yes

Is the statistical analysis and its interpretation appropriate?

Yes

Are sufficient details of the methods and analysis provided to allow replication by others?

Yes

Are the conclusions drawn adequately supported by the results presented in the review?

Partly

Reviewer Expertise:

Animal welfare, behavior, precision livestock farming, thermography, and health.

I confirm that I have read this submission and believe that I have an appropriate level of expertise to confirm that it is of an acceptable scientific standard.

# Reviewer report: Reviewer response for version 2

**DOI:** 10.21956/openreseurope.25698.r73523

**Published:** 2026-05-07

**Authors:** Lorenzo José Fraile

**Affiliations:** 1 Universitat de Lleida, Lleida, Catalonia, Spain

Authors have addressed correctly the questions raised by this reviewer

Are the rationale for, and objectives of, the Systematic Review clearly stated?

Yes

Is the statistical analysis and its interpretation appropriate?

Partly

Are sufficient details of the methods and analysis provided to allow replication by others?

Yes

Are the conclusions drawn adequately supported by the results presented in the review?

Partly

Reviewer Expertise:

Antimicrobial use

I confirm that I have read this submission and believe that I have an appropriate level of expertise to confirm that it is of an acceptable scientific standard.

# Reviewer report: Reviewer response for version 1

**DOI:** 10.21956/openreseurope.25074.r71867

**Published:** 2026-04-17

**Authors:** Naod Thomas Masebo

**Affiliations:** 1 Bologna University, Bologna, Italy

The authors produced an interesting manuscript.

 They wrote a systematic review and meta-analysis article regarding the use of NSAIDs. Most veterinary clinicians use those drugs without being sure whether they are effective or not.  So, this review give an interesting information. 

 The background and objectives, as well as the methodologies, are very clear. 

 My only reservation is the sample size. especially PICO 2, it's a very limited article included. So this part should be considered. 

 The article is written well, and its interesting to read.

Are the rationale for, and objectives of, the Systematic Review clearly stated?

Yes

Is the statistical analysis and its interpretation appropriate?

Yes

Are sufficient details of the methods and analysis provided to allow replication by others?

Yes

Are the conclusions drawn adequately supported by the results presented in the review?

Partly

Reviewer Expertise:

Animal welfare, behavior, and health.

I confirm that I have read this submission and believe that I have an appropriate level of expertise to confirm that it is of an acceptable scientific standard.

# Response

**Published:** 2026-04-23

**Authors:** Karolina Scahill

**Affiliations:** Department of Veterinary Clinical Science, University of Copenhagen, Fredriksberg C, Denmark

Thank you for your positive assessment and for highlighting the sample‑size limitation for PICO 2. We agree. This is reflected in the certainty assessment. We rated down imprecision by three levels for PICO 2 (extremely serious imprecision): the 95% CI crosses three clinical thresholds and encompasses both a large reduction and a large increase in re‑treatment, reflecting very small information size and few events. Under GRADE, this leads to very low certainty, signaling that the direction and magnitude of effect are highly uncertain. This systematic review feeds into a companion ENOVAT guideline, where Evidence‑to‑Decision considerations (feasibility, acceptability, stewardship, and values/preferences) are weighed alongside these effect estimates. The guideline will undergo an open consultation (planned within 1–2 months); we would genuinely welcome your perspective there as well. Because GRADE terminology is relatively new in veterinary medicine, we added a brief clarification in the manuscript to make explicit that the imprecision rating captures sample size and the width of the CI relative to the prespecified clinical thresholds. This does not change the results or conclusions. The following clarification was added to results: *The imprecision domain was rated down three levels because the 95% CI crossed three clinical thresholds; this wide interval reflects the very small information size (only two small RCTs with few events), so the overall certainty of evidence was rated very low (Table 3).*

# Reviewer report: Reviewer response for version 1

**DOI:** 10.21956/openreseurope.25074.r71406

**Published:** 2026-04-06

**Authors:** Lorenzo José Fraile

**Affiliations:** 1 Universitat de Lleida, Lleida, Catalonia, Spain

Authors have carried out an interesting systematic review and meta-analysis for the European Network for optimization of antimicrobial therapy.

 As commented by the authors, the use of non-steroidal anti-inflammatory drugs is widely used in bovine medicine as supportive therapy beside the antimicrobial tratment.

 The methodology used is correct to address the scientific question, but **the reviewer has serious concerns about the final sample size used to address these research questions.**

 For PICO 1, only 17 studies were finally included in the meta-analysis study (Figure 1) and for PICO 2, only 2 studies were finally included (figure 4).

 I understand and agree with the methodology used to have finally these numbers. Neverthelless, authors must review the paper considering the following:

**1.- A sample size calculation must be carried out for addressing PICO 1 and PICO 2 research question to know exactly the potency of the study.**

 2.- I am sure that for PICO 2, the number of required studies are far away from the forme sample size calculation and **this question should be deleted from the research paper. I suggest revising the paper deleting this section.**

 3.- I am quite sure that the number of studies for addressing PICO 1 question would be quite higher than 17. For this reason, authors must highlight that type II error is highly probable in this study. This limitation should be highlighted more in my opinion.

 4.- To finalize, I am wondering that more studies have been carried out by pharmaceutical companies to register combined products (antimicrobial and NSAIDs). It would have been very interesting trying to get this information from pharmaceutical companies to increase the sample size.

 Sincerely yours

 The reviewer

Are the rationale for, and objectives of, the Systematic Review clearly stated?

Yes

Is the statistical analysis and its interpretation appropriate?

Partly

Are sufficient details of the methods and analysis provided to allow replication by others?

Yes

Are the conclusions drawn adequately supported by the results presented in the review?

Partly

Reviewer Expertise:

Antimicrobial use

I confirm that I have read this submission and believe that I have an appropriate level of expertise to confirm that it is of an acceptable scientific standard, however I have significant reservations, as outlined above.

# Response

**Published:** 2026-04-23

**Authors:** Karolina Scahill

**Affiliations:** Department of Veterinary Clinical Science, University of Copenhagen, Fredriksberg C, Denmark

Thank you for your constructive review and for highlighting the sample‑size and grey literature issues. We address each point below and indicate manuscript clarifications to make our GRADE imprecision logic (including information size) more explicit. This systematic review feeds into a companion ENOVAT guideline, where Evidence‑to‑Decision considerations (feasibility, acceptability, stewardship, and values/preferences) are weighed alongside these effect estimates. The guideline will undergo an open consultation (planned within 1–2 months); we would genuinely welcome your perspective there as well.

**Reviewer comment 1: A sample size calculation must be carried out for addressing PICO 1 and PICO 2 research question to know exactly the potency of the study.**

**Response 1:**

 Thank you. The GRADE methodology does not rely on post‑hoc “power” or stand‑alone sample‑size calculations. It does not mean it’s not considered, but it’s reflected in the imprecision domain. Imprecision is judged primarily by how the pooled 95% CI maps onto prespecified clinical thresholds and, secondarily, by information size (participants/events). For PICO 1, the pooled estimate (RR 0.94, 95% CI 0.84–1.05; I²=0%) translates to an absolute effect of 12 fewer per 1,000 (from 31 fewer to 10 more), entirely within the a priori “trivial” range and not approaching any threshold. Under current GRADE guidance, when the entire CI lies within a single range (e.g., trivial), we do not rate down for imprecision and do not invoke the Review/Optimal Information Size (RIS/OIS) checks. RIS/OIS are used when CIs approach or cross thresholds or when a large relative effect appears precise despite very small numbers (to guard against fragile results)—conditions that do not apply to PICO 1. We rated down imprecision by three levels for PICO 2 (extremely serious imprecision): the 95% CI crosses three clinical thresholds and encompasses both a large reduction and a large increase in re‑treatment, reflecting very small information size and few events. Under GRADE, this leads to very low certainty, signaling that the direction and magnitude of effect are highly uncertain. To make this explicit for readers unfamiliar with GRADE, we have added the following clarification to methods: *For imprecision, we used the CI‑based, threshold approach: we rated down by one, two, or three levels if the 95% CI crossed one, two, or three clinical thresholds, respectively. We also considered information size (participants and events); when the CI lay entirely within a single range (e.g., trivial), we did not rate down for imprecision or invoke review information size (RIS)* *. We used RIS only when a CI suggested a moderate/large effect or appeared precise despite very small numbers (* *Zeng et al., 2022* *). We also clarified this point in the results for PICO 2 (very small information size and three thresholds crossed), without changing conclusions.*

**Reviewer comment 2: “I am sure that for PICO 2, the number of required studies are far away from the former sample size calculation and this question should be deleted from the research paper. I suggest revising the paper deleting this section.**

**Response 2: **Thank you for this suggestion. We respectfully disagree that PICO 2 should be deleted. Withholding results because the evidence is sparse would introduce selective reporting (reporting bias) and runs counter to GRADE’s core principle of transparent evidence appraisal. The purpose of a systematic review is to summarize the best available evidence for prespecified questions and then communicate its limitations. For PICO 2 we have done exactly that: we present the pooled estimate and rate down imprecision by three levels (extremely serious), resulting in very low certainty, because the 95% CI crosses three clinical thresholds and the information size is very small (two small RCTs with few events). Our wording is deliberately cautious and explicitly signals that the direction and magnitude of effect are highly uncertain. To help readers unfamiliar with GRADE, we will add a clarification that imprecision reflects both how the CI maps to clinical thresholds and the small information size. The guideline that this SR informs also reflects this uncertainty: it does not endorse NSAID monotherapy, and recommendations are framed in line with the very low certainty of the evidence. We have added the following to the discussion: *For PICO 2, the 95% CI spans both a large reduction and a large increase in re‑treatment, reflecting a very small information size (two small RCTs with few events); we therefore rated imprecision down three levels and judge overall certainty to be very low.*

**Reviewer comments 3 and 4: “(3) I am quite sure that the number of studies for addressing PICO 1 question would be quite higher than 17. For this reason, authors must highlight that type II error is highly probable in this study. This limitation should be highlighted more in my opinion.” “(4) I am wondering that more studies have been carried out by pharmaceutical companies to register combined products (antimicrobial and NSAIDs). It would have been very interesting trying to get this information from pharmaceutical companies to increase the sample size.”**

**Response 3 and 4: **Thank you for these points. We agree that availability and publication biases (including unavailable industry data and non–peer‑reviewed “grey literature”) are important considerations, and we will make this limitation more explicit. On “type II error” for PICO 1: in a meta‑analysis we address the risk of missing a clinically meaningful effect via precision (imprecision) rather than post‑hoc “power.” For PICO 1, the pooled effect and its 95% CI (absolute 12 fewer per 1,000; from 31 fewer to 10 more) lie entirely within the prespecified “trivial” range, heterogeneity was negligible (I²=0%), and sensitivity/subgroup analyses yielded similar estimates. Together, this makes a missed clinically meaningful effect unlikely, though very small effects below the trivial threshold cannot be excluded. On grey literature/industry data: We agree it is a limitaion not including grey literature and recognize this can reduce information size and may introduce availability or publication bias. We assessed publication bias for PICO 1 with a funnel plot (this is only possible when there are over 10 studies), which was approximately symmetric, but acknowledge that such methods cannot rule out missing non–peer‑reviewed or unpublished studies. In veterinary medicine, trial registries are not routinely used, regulatory dossiers are typically not public, and company studies are difficult to identify and obtain systematically—raising a real risk of selective inclusion. For four eligible RCTs with missing extractable counts, we contacted corresponding authors but did not receive data; these trials are summarized descriptively. We will add a paragraph in the discussion acknowledging the limitation of excluding grey literature and the practical barriers to acquiring it in a systematic and unbiased way. If additional eligible data become publicly accessible, we would incorporate them in an update. The following was added to discussion: *Availability and publication bias may affect these results. Our protocol restricted inclusion to peer‑reviewed RCTs; we did not include grey literature. In veterinary medicine, systematic acquisition of unpublished studies is challenging because trial registries are uncommon, regulatory dossiers are typically not public, and company trials are difficult to identify and obtain without introducing selection bias. We explored publication bias for PICO 1 with a funnel plot, which appeared approximately symmetric; however, funnel plots cannot detect studies that were never published or are not peer reviewed, so relevant trials may still have been missed. Although we contacted corresponding authors for four trials lacking extractable counts, no additional data were obtained; these trials are described narratively. This may limit information size and precision, and very small effects below our prespecified “trivial” threshold cannot be excluded.*    
